# Exploring safety culture in the Finnish ambulance service with Emergency Medical Services Safety Attitudes Questionnaire

**DOI:** 10.1186/s13049-021-00960-9

**Published:** 2021-10-12

**Authors:** Anu Venesoja, Veronica Lindström, Pasi Aronen, Maaret Castrén, Susanna Tella

**Affiliations:** 1 South Carelia Social and Healthcare District, Lappeenranta, Finland; 2 Department of Emergency Medicine and Services, University of Helsinki and Helsinki University Hospital, Helsinki, Finland; 3 LAB University of Applied Sciences, Lappeenranta, Finland; 4 Samariten Ambulance, Stockholm, Sweden; 5 Department of Health Promotion Science, Sophiahemmet University, Stockholm, Sweden; 6 Division of Nursing Stockholm, Department of Neurobiology, Care Sciences, and Society, Karolinska Institutet, Stockholm, Sweden; 7 Biostatistics Unit, Faculty of Medicine, University of Helsinki and Helsinki University Hospital, Helsinki, Finland; 8 University of Eastern Finland, Kuopio, Finland

**Keywords:** Safety culture, Safety climate, EMS-SAQ, Prehospital, Patient safety, Survey, Cross-sectional, Psychometric test

## Abstract

**Background:**

Emergency Medical Services (EMS) is, by its nature, a challenging context that may create risks for both patients and employees. It is also known that an organisation’s safety culture has an influence on both patient and employee safety. Finnish EMS organisations lack knowledge of how their safety culture is perceived by their employees.

**Aim:**

This study aims to test the psychometric properties of the Emergency Medical Services Safety Attitudes Questionnaire (EMS-SAQ) in a Finnish EMS setting. We also explore the connections between individual- and organisation-based characteristics and safety attitudes in the Finnish EMS.

**Methods:**

A cross-sectional survey study design was used. The EMS-SAQ was used to collect data via social media. The instrument measures six domains of workplace safety culture: safety climate, teamwork climate, perceptions of management, job satisfaction, working conditions and stress recognition. The 5-point Likert scale was converted to a 100-point scale and mean ≥ 75 was dichotomized as a positive. Confirmatory factor analysis (CFA) was carried out to validate the EMS-SAQ in a Finnish setting. Other results were analysed by using non-parametric tests.

**Results:**

327 responses were included in the analysis. CFA showed that the total EMS-SAQ model had acceptable goodness-of-fit values in the Finnish EMS setting. Total mean scores for each safety culture domain were identified non-positively (mean score < 75); safety climate 60.12, teamwork climate 60.92, perceptions of management 56.31, stress recognition 64.55, working conditions 53.43 and job satisfaction 70.36. Higher education was connected to lower job satisfaction and the teamwork climate within the individual characteristics. All organisation-based characteristics caused at least one significant variation in the safety culture domain scores. Working area significantly affected (*p* < 0.05) five out of the six safety culture domain scores.

**Conclusions:**

The EMS-SAQ is a valid tool to evaluate safety culture among the Finnish EMS organisations; it offers a novel method to evaluate safety and patient safety within the Finnish EMS organisations. According to the findings, the organisation-based characteristics more likely had an impact on safety attitudes than did the individual-based characteristics. Therefore, it is suggested that the Finnish EMS organisations undertake safety culture development at the organisational level.

**Supplementary Information:**

The online version contains supplementary material available at 10.1186/s13049-021-00960-9.

## Background

EMS are defined as safety–critical organisations similar to organisations such as chemical and nuclear installations, military field units and hospital trauma units [1]. Ensuring safety plays a vital role in all of these organisations. Being a safety–critical organisation means that a risk exists for people being injured or dying as a result of performing their tasks [2]. An EMS organisation is considered to be safety–critical even though not all tasks contain an immediate risk for the professionals or the patients; therefore, it is important to explore the EMS safety culture.

The World Health Organization (WHO) has defined the term safety culture as *the product of individual and group values, attitudes, perceptions, competencies, and patterns of behavior that determine the commitment to, and the style and proficiency of, an organization’s health and safety management* [3]*.* Regardless of the WHO definition, the terms *safety culture* and *safety climate* have usually been used synonymously; researchers have even discussed the terms’ differences and similarities. The literature has described the safety culture as being less flexible and more complex than the safety climate. The safety climate is measurable on several different levels, for example, the individual, team, unit, organisation and environment levels [4, 5]. According to Flin et al., safety climate studies are regarded as attitude studies [6]. Despite the differences between those two terms, we use the term safety culture for clarity in this study and include safety climate in safety culture.

One of the key areas for improving safety in healthcare focuses on building a positive safety culture [7]. A safety culture formulates situation awareness, communality and cooperation between an organisation, its supervisors/managers and employees [8]. The leaders have an important role in fostering safety and team performance [9]. For example, in a health-care setting, management’s commitment to safety is related to teamwork [10]. National guidance and organisation management and leadership in the emergency medical service (EMS) setting play the most significant roles in promoting a safety culture. EMS personnel attitudes toward the organisation, the work environment, teams and staff are components of a safety culture [11].

The WHO has underlined the importance of a safety culture when organisations develop patient safety. It is described that enhancing safety culture in health-care services may have a positive impact on patient safety. Effective leadership and a supportive culture are essential for improving safety. Employees’ safety and patient safety are combined for their well-being at work and for employees’ output. The working and caring environment should encourage the professionals and patients to speak up freely without fear of retribution. People want to report risks and safety incidents in order to learn from them in that kind of environment. It is important to create an environment where people understand that incidents are caused largely by system failures rather than by individuals [12]. Moreover, employees’ well-being, safety and patient safety should be seen as a result of the organisation’s safety culture [4].

Safety culture in the EMS setting has previously been studied by using the Emergency Medical Services Safety Attitudes Questionnaire (EMS-SAQ) [13–15]. There is some evidence that the safety outcomes and the safety culture experienced by EMS workers, patients and providers are interrelated. Weaver et al. found that respondents who reported injuries evaluated their safety culture lower than those who did not report injuries. Respondents who reported an error or an adverse event (AE) also evaluated their safety lower than those who did not. Respondents who reported safety-compromising behavior evaluated their safety culture as lower [16]. However, a limited number of safety culture studies have been performed on EMS worldwide, and the EMS-SAQ has never been used to study the safety culture in the Finnish EMS. Therefore, this study aims to test the psychometric properties of EMS-SAQ in a Finnish EMS setting; in addition, we explore the connections between individual- and organisation-based characteristics and safety attitudes in the Finnish EMS setting.

## Methods

### Study design and instrument

We used a cross-sectional survey design and collected the data using the EMS-SAQ (see Additional File 1). We enquired about receiving permission to use the EMS-SAQ instrument from Ph.D. Patterson. Patterson et al. developed the EMS-SAQ by modifying the Intensive Care Unit Safety Attitude Questionnaire (ICU-SAQ) to be suitable to the EMS setting [14]. Based on the ICU-SAQ [17], the EMS-SAQ has 30 core items. Those items characterise six safety culture domains: (1) safety climate (seven items), (2) job satisfaction (five items), (3) perceptions of management (four items), (4) teamwork climate (six items), (5) working conditions (four items) and (6) stress recognition (four items). Respondents answer every question by using a 5-point Likert scale (1 = Disagree strongly, 2 = Disagree slightly, 3 = Neutral, 4 = Agree slightly, 5 = Agree strongly).

The psychometric-tested Finnish version of the EMS-SAQ comprises 59 questions. Fifty items concern the safety culture in the EMS, four items cover the respondents’ characteristics (age, gender, education level, working experience), and five questions concern organisation-based characteristics (position type, employment status, shift length, employer status and which catchment area for highly specialized medical care the participant works in). Additional file 1 presents the questionnaire we used. We followed the Checklist for Reporting Results of Internet E-Surveys (CHERRIES) to enhance the quality and transparency of this web survey study [18].

### EMS-SAQ translation into Finnish

Double blind forward and back translations were used to translate the original English version into Finnish [19]. The translated version was then evaluated by four EMS personnel to pretest the clarity of the EMS-SAQ Finnish version. One question’s word order was adjusted based on their oral feedback to the researcher.

### Setting

Finland has 21 different hospital districts responsible for organizing the EMS. They can provide the EMS by themselves, or they can purchase the EMS from another party, for example, from a rescue department, other hospital districts or the private sector [20]. The EMS include advanced level and basic level ambulances. Advanced level ambulances are manned with least one prehospital emergency care nurse or a registered nurse (RN) with 30 European Credit Transfer and Accumulation System (ECTS) study points of prehospital emergency care studies (Bachelor’s level education). The basic level ambulance must be manned by at least one person who is a health-care professional with a vocational education that includes emergency medical training, i.e., Emergency Medical Technician (EMT), practical nurse, firefighter/rescue worker. A master’s degree is not a requirement in any of the work tasks in a Finnish EMS [21]

Every hospital district should have an EMS officer and an EMS medical director in accordance with national regulations. The EMS officer is an operational supervisor of the shift and participates as a leader in challenging tasks and mass casualty situations. The EMS medical directors’ responsibilities are to prepare service standard decisions, participate in preparedness planning with other authorities, write out EMS prescriptions, give guidance to the emergency response centers regarding how to dispatch the EMS units and to confirm the EMS workers’ treatment obligations [21]. The EMS organisations also have managers and/or supervisors who are responsible for human resource management, for example. There are presently no regulations concerning EMS supervisors’ or managers’ educational levels.

Five university hospitals are responsible for providing all highly specialised medical care services required within their catchment areas [20]. The university hospital areas coordinate the EMS performance in their hospital districts regarding the local special features, develop the EMS together nationally with the other university hospital areas, and promote scientific research. Furthermore, all university hospital areas are responsible for having an EMS medical doctor available 24/7 in least one location in their area. The physician’s responsibility is to lead the EMS operational situations in the area together with the EMS officers [21]. Table 1 presents Finland’s geographical size, population, number of full-time EMS personnel and annual number of EMS calls (total and between the catchment areas).

**Table 1:** Finland’s geographical size, population, number of full-time Emergency Medical Service (EMS) personnel and annual number of EMS calls (total and between the catchment areas)

Geographical size (km^2^)*	390,909 km^2^				
People live in Finland**	5,s525,292				
Full-time EMS workers	3898				
- Advanced	2611				
- Basic	1287				
EMS calls (2019)**	811 385				
Catchment area for highly responsive care	HUH^†^	TYKS^††^	TAYS^†††^	KUH^††††^	OYS^†††††^
Areas’ geographical size (km^2^) *	36,642 km^2^	62,800 km^2^	37,073 km^2^	78,268 km^2^	176,126 km^2^
People living in the area (31.12.2019) **	2,188,253	898,300	901,358	800,498	736,883
Full-time EMS workers in the area	814	699	731	812	842
Advanced	675	444	444	511	537
Basic	139	255	255	301	305
EMS calls (2019)**	268,365	117,268	130,833	154,562	140,357

^†^ Helsinki University Hospital

^††^Turku University Hospital

^†††^Tampere University Hospital

^††††^Kuopio University Hospital

^†††††^Oulu University Hospital

*NLS National land survey of Finland

**Sotkanet.fi Statistical information on welfare and health in Finland

### Data collection, participants and sample size

Data were collected between 5th of December 2019 to 5th of January 2020 by sharing the web-based survey link (Webropol®) to the questionnaire in social media (Facebook® and Instagram®). Social media have been previously shown to be an effective way to collect data in health, medical and social research [22, 23]. The link was posted to the one Facebook® group and the two Facebook® pages with links to Instagram®. Members in those groups are mainly EMS professionals, or they have a special interest in EMS, or they are EMS stakeholders. However, not everyone who is active in these social media channels works in the EMS field. Therefore, we informed them that this questionnaire is intended for those people who are working full time or part time in EMS. No incentives were distributed to the participants.

A data protection statement and information about the study’s purpose were shared to the participants with the questionnaire. Answering the survey was considered consent to participate in the study. Therefore, the need for written informed consent was waived because of the study design. Answering the survey was voluntary, and the participants had the right and possibility to withdraw from the study by suspending their answering or by selecting not to send the answers [24–26].

The sample size calculation for multiple factors analysis research should be at least 10 times the number of items [27, 28]. Therefore, the minimum acceptable sample size in this study was 300.The invitation to participate in the study reach 6196 people, 954 of whom opened the link to the questionnaire; 418 started to respond and a total of 333 people completed the survey. The response rate can be calculated by dividing the number of people completing the questionnaire by those who viewed it [29]. It is reasonable to assume a response rate of 34.9% using this calculation.

### Statistical analysis

Confirmatory factor analysis (CFA) was carried out to validate the EMS-SAQ in a Finnish setting. Missing values were imputed with the median, which is acceptable when only a small number of values are missing [30]. Missingness varied from 0% (22 questions) to 0.9% (“Q18”). Normality was screened using normality plots of histogram and kurtosis/skewness values [31]. We found in 18 variables indications of nonnormality (kurtosis or skewness values larger than ± 1). The maximum likelihood (ML) with 1000 bootstrapped replications was used because of the nonnormality [27]. We used comparative fit index (CFI); Tucker–Lewis index (TLI)/non-normed fit index (NNFI) and the root-mean-square error of approximation (RMSEA) to assess fit. Following the recommendation by Hair [32], the criteria were set to evaluate the adequacy of the model as CFI > 0.90, NNFI > 0.90, and RMSEA < 0.07. The modification indices (MI) were examined to identify any additional adjustments. The reliability level was set at ≥ 0.7 using Cronbach’s alpha [33].

The 5-point Likert scale was converted to a 100-point scale, where 0 = Disagree strongly, 25 = Disagree slightly, 50 = Neutral, 75 = Agree slightly, 100 = Agree strongly. Two questions (SC4 *In this EMS agency it is difficult to discuss errors* & TWC2 *At this EMS agency, it is difficult to speak up if I perceive a problem with patient care*) were reverse coded in the analysis to match the other questions. In line with prior studies, [14, 15, 17] dichotomized the safety culture domain scores as a “positive” (≥ 75) and “non-positive” (< 75). Therefore, the respondents should have answered with an average of Agree Slightly or higher to count as positive. The connections between individual- and organisation-based characteristics and safety attitudes in the Finnish EMS were tested by using appropriate nonparametric tests. The statistical significance level was set at 0.05. P-values have been adjusted by the Bonferroni correction for multiple tests. SPSS AMOS 25.0 was used for the CFA, and IBM SPSS version 25.0 for other statistical analyses.

## Results

### Respondents

The total sample size was 333. The final sample size was 327 after omitting six responses because of divergent shift length (three-shift, day job). Table 2 presents the respondents’ demographic information.

**Table 2:** Characteristics of participants

Characteristic	Total N = 327
Gender	324 (100%)
Female	161 (49.69%)
Male	163 (50.31%)
NA	3
Age	273 (100%)
£25	23 (8.42%)
26–30	78 (28.57%)
31–35	71 (26.00%)
36–40	53 (19.41%)
41–45	30 (10.99%)
^3^46	18 (6.59%)
NA	54
Education level	327 (100%)
Master’s	50 (15.29%)
Bachelor’s	225 (68.81%)
Vocational *	46 (14.07%)
Other	6 (1.83%)
Working experience	327 (100%)
£5 years	110 (33.64%)
6–10 years	113 (34.56%)
11–15 years	53 (16.21%)
^3^15 years	51 (15.60%)
Position type	325 (100%)
Advanced level	264 (81.23%)
Basic level	61 (18.77%)
NA	2
Employment status	327 (100%)
Full-time	295 (90.21%)
Part-time	32 (9.79%)
Shift type	327 (100%)
24-h shifts	131 (40.06%)
Two shift	181 (55.35%)
Mix (24 h + 12 h and/or 8 h)	15 (4.59%)
Affiliation	327 (100%)
Health care district	163 (49.85%)
Fire department	119 (36.39%)
Private	47 (14.37%)
Catchment area for highly responsive care	327 (100%)
Helsinki University Hospital	112 (34.25%)
Turku University Hospital	41 (12.54%)
Tampere University Hospital	50 (15.29%)
Kuopio University Hospital	63 (19.26%)
Oulu University Hospital	61 (18.65%)

*Vocational educated EMS personnel = EMT, practical nurse, firefighter/ rescue worker

### Finnish EMS-SAQ validity and reliability results

**Fig. 1:**
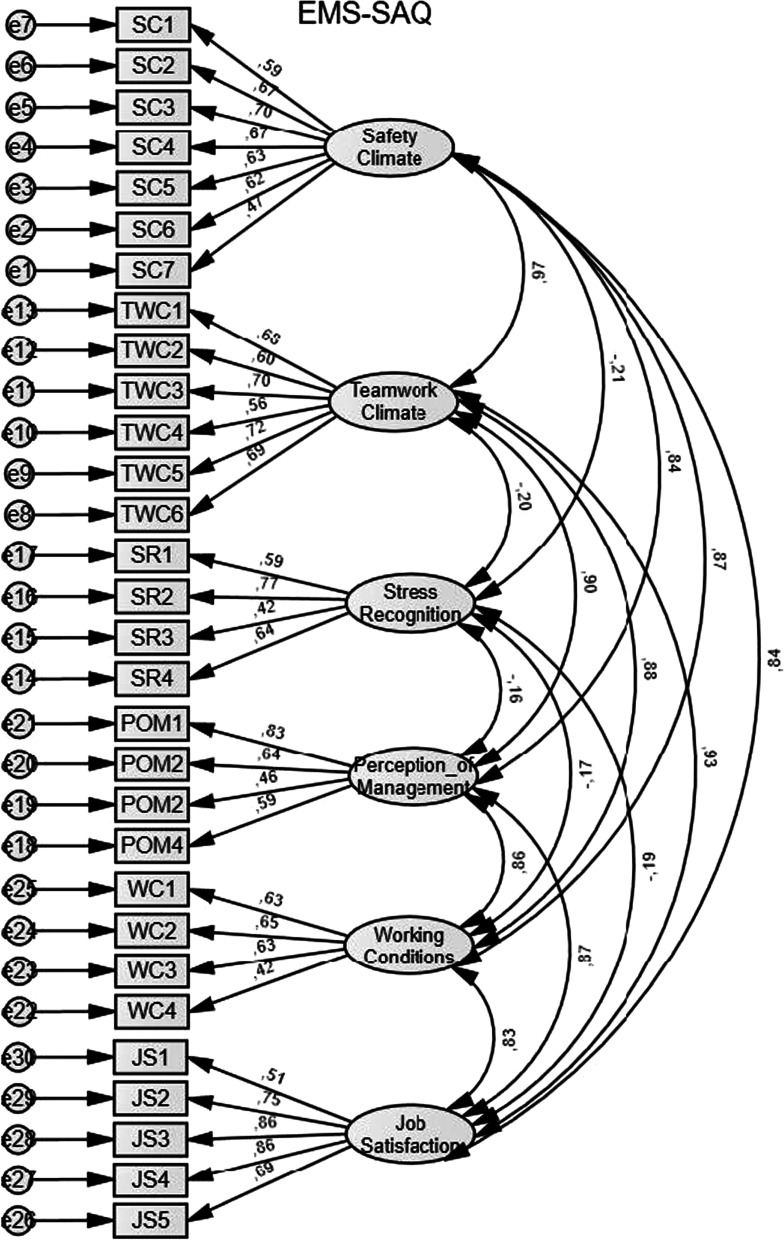
Original model with correlations between the safety culture domains and items factor loadings

According to the chi-square test of model, fit was significant (χ^2^ = 828.471, degrees of freedom (df) = 390, p = 0.000). RMSEA was 0.059, also suggesting good fit, but the results from CFI (0.896) and TLI (NNFI) (0.884) indicated a slight lack of fit. Factor loadings ranged from 0.86 to 0.42. The correlations between five out of the six factors were high (0.84—0.97). Stress recognition has a negative correlation among the other factors (-0.16 to -0.21). Figure 1 presents the original CFA model with correlations between the safety culture domains and items factor loadings.

A better fit was gained by adjusting the model. Based on modification indices (M.I.), two acceptable error terms (questions SC1 *I would feel safe being treated by this EMS agency as a patient* vs SC4 *In this EMS agency it is difficult to discuss errors*, and WC1 *This EMS agency does a good job of training new personnel* vs WC3 *Trainees in my discipline are adequately supervised*) were allowed to correlate. After adjustments, model fit results were: χ^2^ 790.333, df = 388 p = 0.000, CFI = 0.904, TLI (NNFI) = 0.893 and RMSEA = 0.056.

Total model Cronbach’s alpha was 0.871. Single domains Cronbach’s alpha ranged between 0.851 (Job satisfaction) to 0.660 (Working conditions). Table 3 presents all of the safety culture domains’ reliability results.

**Table 3:** Variation in safety culture domain scores between the respondent’s characteristic

	Safety climate a = 0.810	Teamwork climate a = 0.821	Perceptions of management a = 0.726	Stress recognition a = 0.689	Working conditions a = 0.660	Job satisfaction a = 0.851
Mean (standard deviation)						
Total (n = 327)	60.04 (18.40)	60.95 (18.40)	56.19 (22.10)	64.62 (18.40)	53.40 (20.30)	70.26 (19.42)
Gender (n = 324)	NS	NS	NS	NS	NS	NS
Female (161)	59.23 (18.11)	60.43 (17.37)	58.15 (20.86)	66.61 (17.86)	53.42 (21.37)	70.99 (18.18)
Male (163)	60.71 (18.79)	61.17 (19.31)	54.33 (23.11)	62.85 (20.69)	54.33 (23.11)	69.45 (20.65)
Age (n = 273)	NS	NS	NS	NS	NS	NS
≤ 25 years (23)	64.60 (13.79)	62.32 (14.18)	54.62 (19.24)	69.57 (14.99)	52.67 (17.64)	71.52 (15.11)
26–30 years (78)	57.69 (17.90)	60.04 (17.59)	58.57 (20.42)	62.58 (19.88)	51.84 (19.92)	71.03 (16.74)
31–35 years (71)	57.75 (19.22)	59.27 (17.27)	53.87 (20.13)	65.05 (17.60)	52.11 (20.15)	67.75 (19.49)
36–40 years (53)	58.76 (18.30)	61.40 (19.87)	52.59 (22.41)	67.57 (22.44)	52.48 (23.04)	68.21 (22.41)
41–45 years (30)	62.86 (18.68)	62.64 (17.80)	53.54 (25.93)	66.04 (17.96)	55.63 (18.01)	70.50 (17.63)
≥ 46 years (18)	63.29 (22.25)	56.25 (21.87)	59.72 (24.65)	61.11 (24.96)	56.25 (22.38)	70.83 (21.09)
Education level (n = 327)	NS	*p* = 0.009	NS	NS	NS	*p* = 0.021
Master’s (50)	56.92 (19.70)	53.75 (20.40)	51.13 (22.75)	66.00 (20.95)	52.13 (20.07)	63.80 (20.57)
Bachelor’s (225)	59.92 (18.33)	61.69 (17.88)	56.67 (22.30)	65.75 (18.65)	52.69 (20.01)	70.80 (19.33)
Vocational (46)	64.83 (16.72)	66.49 (16.03)	60.46 (20.16)	58.29 (20.84)	57.74 (22.25)	75.76 (16.05)
Other (6)	53.57 (18.49)	50.69 (20.31)	47.92 (17.97)	59.37 (19.26)	57.29 (16.96)	61.67 (24.63)
Working experience (n = 327)	NS	NS	NS	NS	NS	NS
≤ 5 years (110)	62.18 (16.59)	62.84 (15.76)	59.89 (21.07)	66.48 (18.66)	51.88 (19.07)	73.95 (14.89)
6–10 years (113)	57.78 (19.50)	61.69 (19.29)	56.14 (20.85)	62.50 (20.20)	55.59 (20.16)	69.25 (21.44)
11–15 years (53)	59.30 (17.33)	55.82 (19.05)	49.88 (22.38)	64.74 (20.59)	50.35 (19.59)	64.62 (20.96)
> 15 years (51)	61.20 (20.47)	60.54 (20.44)	54.90 (25.47)	65.19 (18.21)	55.02 (23.59)	70.39 (20.61)
Position type (n = 325)	NS	NS	NS	NS	*p* = 0.032	NS
Advanced level (264)	59.02 (18.96)	60.21 (18.66)	55.75 (22.14)	65.63 (19.22)	52.18 (20.45)	69.45 (20.11)
Basic level (61)	64.29 (15.50)	63.93 (17.31)	57.79 (22.35)	60.25 (20.19)	58.50 (19.20)	73.61 (16.10)
Employment status (n = 327)	*p* = 0.020	NS	*p* = 0.037	NS	NS	NS
Full-time (295)	59.23 (18.47)	60.52 (18.52)	55.36 (22.06)	64.96 (19.32)	52.82 (20.42)	69.68 (19.72)
Part-time (32)	67.52 (16.08)	64.84 (16.96)	63.87 (21.34)	61.52 (20.58)	58.79 (18.57)	75.63 (15.75)
Shift type (n = 327)	NS	*p* = 0.011	*p* = 0.001	NS	NS	*p* = 0.008
24-h shifts (131)	61.64 (18.89)	64.53 (19.24)	61.50 (23.59)	64.27 (19.24)	54.91 (21.41)	73.74 (20.01)
Two shift (181)	59.18 (18.21)	58.63 (17.80)	52.45 (20.11)	63.16 (19.72)	52.52 (19.96)	67.98 (18.91)
Mix (24 h + 12 h and/or 8 h) (15)	56.43 (16.04)	57.50 (15.12)	55.00 (23.76)	73.33 (16.61)	50.83 (13.34)	67.33 (16.78)
Affiliation (n = 327)	NS	NS	*p* = 0.008	NS	*p* = 0.002	NS
Health care district (161)	59.58 (18.01)	59.16 (18.12)	52.68 (22.13)	65.92 (17.51)	49.57 (20.01)	68.11 (19.46)
Rescue department (119)	59.54 (18.84)	63.06 (18.31)	58.25 (21.40)	64.34 (21.77)	58.66 (19.88)	72.90 (18.67)
Private (47)	62.84 (18.73)	61.70 (19.32)	63.03 (21.96)	60.90 (19.35)	53.19 (19.76)	70.96 (20.68)
Catchment area for highly responsive care (n = 327)	*p* = 0.023	*p* = 0.006	*p* = 0.000	NS	*p* = 0.012	*p* = 0.002
Helsinki University Hospital (112)	62.56 (18.06)	59.44 (19.07)	53.07 (23.22)	65.85 (19.39)	52.79 (19.97)	69.06 (20.41)
Turku University Hospital (41)	59.58 (20.87)	64.23 (17.20)	59.76 (21.65)	66.62 (19.04)	52.74 (24.61)	74.51 (19.33)
Tampere University Hospital (50)	57.71 (18.17)	59.33 (18.63)	61.38 (20.31)	66.00 (21.32)	50.50 (18.07)	69.70 (21.01)
Kuopio University Hospital (63)	54.54 (16.18)	56.75 (14.73)	45.44 (17.93)	59.62 (18.23)	50.10 (17.19)	64.84 (15.63)
Oulu University Hospital (61)	63.29 (18.59)	67.14 (19.75)	66.39 (19.97)	65.06 (19.17)	60.76 (21.23)	75.65 (18.49)

All *p* values counted with non-parametric tests (Mann–Whitney-U and Kruskal–Wallis)

*p* values adjusted by the Bonferroni correction for multiple tests

A 100-point scale: 0 = Disagree strongly, 25 = Disagree slightly, 50 = Neutral, 75 = Agree slightly, 100 = Agree strongly (^3^75 = positive)

### Connections between individual and organisation-based characteristics and safety attitudes

**Fig. 2:**
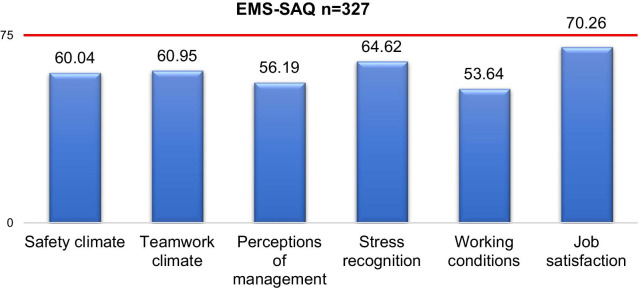
Total mean scores for each safety culture domains

For the study dimensions (safety climate, teamwork climate, perceptions of management, stress recognition, working conditions and job satisfaction), means and standard deviation scores were calculated for age, gender, education, working level, working experience, employment status, shift length, employer status and catchment area for highly specialised medical care (see Table 3). Total mean scores for each safety culture domain were identified non-positively (< 75), see Fig. 2. A percentage of positive (≥ 75) responses were also calculated. The total percentages for positive responses for each safety culture domain were safety climate 25.4, teamwork climate 26.9, perceptions of management 27.2, stress recognition 37.3, working conditions 18.7 and job satisfaction 51.1 (see Additional File 2).

Gender, age or working experience within the individual characteristics (gender, age, education level, working experience), did not affect variations in safety culture scores. Higher education was connected to a lower teamwork climate and job satisfaction. Master’s educated EMS personnel had a lower score in teamwork climate (p = 0.012) and job satisfaction (*p* = 0.022) than vocational educated personnel in the pairwise comparisons. None of the individual characteristics had a significant impact on the percentage of positive responses.

Organisation-based characteristics (position type, employment status, shift type, affiliation, working area) were connected to significant variations in safety culture domain scores and the percentage of positive responses on each domain. EMS organisations’ different shift types (24 h/ two-shift/ mix) significantly affected EMS personnel’s views in the teamwork climate, perceptions of management and job satisfaction domain scores. EMS personnel in the pairwise comparisons who work in a two-shift system had a significantly lower score in teamwork climate (*p* = 0.012), perceptions of management (*p* = 0.001) and job satisfaction (*p* = 0.008) than those who work in the 24 h shifts.

EMS personnel’s affiliations (health care district/rescue department/private) had a significant impact on their perceptions of management and working conditions domain scores. Perceptions of management scores in the pairwise comparisons were significantly lower (mean 52.68 vs. 63.03, *p* = 0.015) for EMS personnel who work in the health-care districts than for those who work in private companies. EMS personnel who work in the health-care districts scored their working conditions lower (mean 49.57 vs. 58.66, *p* = 0.001) than did those who work in rescue departments.

The working area organisational characteristic created the significant variation between the safety culture domain scores and the percentage of positive responses. The safety climate domain scores had significant variations between the Kuopio University Hospital Districts area and in the Helsinki (*p* = 0.033) or Oulu (*p* = 0.046) University Hospital Districts areas in the pairwise comparisons. The teamwork climate domain scores had significant differences between the Kuopio and Oulu University Hospital Districts areas (*p* = 0.005) and between the Helsinki and Oulu University Hospital Districts areas (*p* = 0.043). The perceptions of management scores vary significantly between the Kuopio and Turku University Hospital Districts areas (*p* = 0.015), the Kuopio and Tampere University Hospital Districts areas (*p* = 0.001), the Kuopio and Oulu University Hospital Districts areas (*p* = 0.000) and the Helsinki and Oulu University Hospital Districts areas (*p* = 0.002). Working conditions domain scores had significant differences between the Kuopio and Oulu University Hospital Districts areas (*p* = 0.013) and the Tampere and Oulu University Hospital Districts areas (*p* = 0.046). Job satisfaction was lower for EMS personnel who work in the Kuopio University Hospital Districts area than those who work in the Turku University Hospital Districts area (*p* = 0.019) or the Oulu University Hospital Districts area (*p* = 0.002).

## Discussion

This is the first time, to the best of our knowledge, when this EMS-SAQ instrument has been validated outside of North America. We used the same set of fit indices as Patterson et al. to keep the studies comparable [14, 15]. The CFA results show that the model fit was not entirely optimal with this data collected from Finnish EMS workers. However, the model fit was better with a minor adjustment [32]. On the other hand, reliability scores were close or above the acceptable level (0.7), thus demonstrating good internal consistency of the total model and the individual domains. Therefore, we conclude that the Finnish translation of EMS-SAQ is an appropriate tool for studying safety culture in this EMS setting, even if our CFA results were not as good as in previous EMS-SAQ studies [14, 15]. There also seems to be some variation in SAQ’s CFA results in other health-care settings and different countries [34–37], and it should be noted that cultural differences could affect the CFA results [38–40] in safety culture studies.

Correlations between the safety culture domains were in the same direction as in Sexton et al.’s original SAQ version [17]. The strong correlations shown in the CFA results between the five out of six safety culture domains raise the question of whether it is possible that we could keep strong correlations by improving only one domain with an intervention. Does it have an impact on other safety culture domains if organisations can develop one safety culture domain? For example, if organisations develop their management policies, will the teamwork climate or working condition experience improve at the same time? Measuring EMS personnel’s safety attitudes makes it possible to identify the safety culture’s strengths and weaknesses and utilize this information for safety culture development. Therefore, we think that this EMS-SAQ tool could help the organisations compare their safety culture before and after the interventions.

Organisation-based characteristics caused most of the significant variations in safety culture domain scores, so it is reasonable to think that the safety culture should be evaluated and developed at the organisational level. Previous research has shown that the organisation climate predicts the team-level climate, which has an impact on behavior [41, 42]. Management commitment to safety was positively correlated with teamwork in a hospital setting [10]. Other than that, another study conducted in Finland stated that the social environment and the paramedics’ role in the working community have a significant role establishing a safety culture in an EMS organization [11]. It would improve the safety culture, more specifically the teamwork climate, if EMS teams in the field could create a psychologically safe [43] teamwork environment. As an example, a previous study has shown improvements in safety climate and teamwork climate scores after interventions. That same study also reported that harm, serious safety events and severity-adjusted hospital mortality has decreased in all hospitals [44].

It was interesting to find that the more highly educated EMS personnel evaluated the teamwork climate and job satisfaction lower than did the less educated EMS personnel. All in all, higher-educated EMS personnel evaluated five out of the six safety culture domain scores (three of them *p* =  >0.05) lower than less-educated EMS personnel. These findings were not in line with previous studies conducted within EMS-SAQ [15, 16]. The reason is unclear, but it seems that the higher-educated EMS personnel recognise stress slightly better than the less-educated EMS personnel, which was a similar finding in previous studies [15, 16]. The lowest scores for five out of the six safety culture domains could indicate that the higher-educated EMS personnel have lower occupational callings that affect safety climate and safety behavior [12]. In parallel, it is relevant to note that the wellbeing and safety of healthcare providers is also related to patient safety [45, 46]. It is described that education level has a positive influence on the accuracy of primary diagnoses made by paramedics who have a bachelor-level degree in prehospital nursing [47] and on the reduction of the mortality and complication rates for patients in surgical wards [48]. Based on this previous knowledge, an assumption is that higher-educated EMS personnel have better capabilities to evaluate critically all the factors affecting safety culture in the EMS. Furthermore, the lowest scores from the higher-educated personnel in five out of six safety culture domains have not, to our knowledge, been shown before. Therefore, this phenomenon needs more research in the future not only in the EMS setting but also in other healthcare settings. This is especially true because studies conducted with other versions of SAQ have rarely used education level as a background variable.

It is important that organisations evaluate not only direct measures (errors and adverse events) of patient safety but also other subareas of patient safety [7]. Safety culture has clear connections to managements’ actions and perceived perceptions of the safety culture [8, 41]; thus, it is crucial to evaluate how safety culture in the EMS setting affects patient safety from that direction. Employee working commitment has an impact on the safety climate, and the most important factors that impact on working commitment are the supervisors’ ability to recognize a growing climate and the extent to which they allow the climate to grow [41, 42]. The EMS-SAQ should be seen as one tool for EMS supervisors when they evaluate patient safety and plan development measures for their organisations.

## Limitations

This study has some limitations. Data collection via social media could be considered a limitation. Reducing the risk of receiving responses from participants outside of our target group meant that we did not share any incentives with the participants. Using social media and a web-based survey as data collection methods could constitute a risk for selection biases, which could limit the results’ generalisability [29]. This study’s selection biases occur as a potentially non-representative nature of the social media population and a possible self-selection bias. Our study’s self-selection bias means that respondents could be those who are interested in or maybe concerned with this topic. However, it has been stated that social media and, more specifically, Facebook, have several advantages, for example costs, time and a snowball effect, when collecting data [22, 23]. Also, previous results suggest that data collected via Facebook is similarly representative to data collected via more traditional methods [23]. The aim of this study was to gain an overview of the prevailing safety culture in Finnish EMS rather than to compare the safety culture between the organisations. Therefore, data collection via social media can be seen as a valid data collection method.

Otherwise, this study has limitations similar to other studies that have collected data via web-based survey [49, 50]. This study has one important selection bias. Firefighters also work in ambulances in most rescue department-based EMS systems. However, a limited number of respondents identified as firefighters, yet firefighters usually work most of the time in rescue units, which could cause a different kind of bias to the results.

Another limitation is that we do not know exact organisational information about the respondents. Therefore, this study is not directly generalisable to the organisational level. However, even though we received responses from all over Finland, one might think that the results’ generalisability could be limited because of the data collection method. This study, despite its limitations, presents one of the important dimensions of the prevailing safety culture in Finnish EMS organisations and in EMS settings with similar prerequisites, but further research is needed.

## Conclusions

The EMS-SAQ is a valid tool to evaluate safety cultures in Finnish EMS settings. The EMS-SAQ can offer a new method to evaluate safety and patient safety in EMS settings before and after interventions (for example, large software changes, organisational changes, or changes in the working hours) on both the national and organisational levels. The overview of the Finnish EMS personnel’s safety attitudes was identified non-positively. There was a significant variation in the safety culture scores. Of this variation, the organisation-based characteristics more likely had an impact on safety attitudes than did the individual characteristics. Therefore, the safety culture in the Finnish EMS setting requires more evaluation and development at the organisational level. Enhancing the organisational-level development of the safety culture requires national-level attention by EMS managers and supervisor education.

## Supplementary Information

**Additional file 1**. EMS-SAQ and questions/ domain [14, 15]

**Additional file 2**. Percentage of positive responses between the respondent’s characteristic

## Data Availability

The full datasets used and analysed during the current study and the translated version of the EMS-SAQ are available from the corresponding author on reasonable request.

## Abbreviations

WHO: World Health Organization
EMS: Emergency medical services
EMS-SAQ: Emergency Medical Services Safety Attitudes Questionnaire
AE: Adverse event
ICU-SAQ: Intensive care unit safety attitudes questionnaire
SAQ: Safety attitudes questionnaire
ECTS: European Credit Transfer and accumulation System
RN: Registered nurse
EMT: Emergency medical technician
CFA: Confirmatory factor analysis
CFI: Comparative fit index
TLI: Tucker–Lewis index
NNFI: Non-normed fit index
RMSEA: Root-mean-square error of approximation

## References

[1] Dov Z (2008). Safety climate and beyond: a multi-level multi-climate framework. Saf Sci.

[2] Oxford english dictionary. Oxford University Press, 2020 http://www.oed.com/viewdictionaryentry/Entry/11125. Accessed 4 Dec 2020

[3] WHO World Alliance for Patient Safety, Geneva; 2010. Conceptual framework for the international classification for patient safety version 1.1: final technical report. https://apps.who.int/iris/handle/10665/70882. Accessed: 4 Dec 2020

[4] Flin R (2007). Measuring safety culture in healthcare: a case for accurate diagnosis. Saf Sci.

[5] Guldenmund FW. Understanding and exploring safety culture. Technical Univer- sity of Delft. Faculty of technology, policy and management. Safety science group. Doctoral thesis. Uitgeverij Boxpress, Oisterwijk. 2010.

[6] Cox S, Flin R (1998). Safety culture: philosopher's stone or man of straw?. Work Stress.

[7] World Health Organization; 2016. Patient engagement. https://apps.who.int/iris/handle/10665/252269. Accessed: 4 Dec 2020.

[8] Sætrevik B, Hystad SW (2017). Situation awareness as a determinant for unsafe actions and subjective risk assessment on offshore attendant vessels. Saf Sci.

[9] Künzle B, Kolbe M, Grote G (2010). Ensuring patient safety through effective leadership behaviour: a literature review. Saf Sci.

[10] McGonagle AK, Essenmacher L, Hamblin L, Luborsky M, Upfal M, Arnetz J (2016). Management commitment to safety, teamwork, and hospital worker injuries. J Hosp Admin.

[11] Venesoja A, Windahl T, Hänninen S, FT NN. Ensihoitajien käsityksiä ensihoidon turvallisuuskulttuuriin vaikuttavista tekijöistä [Paramedics’ perceptions of the factors affecting the prehospital emergency care safety culture]. Tutkiva Hoitotyö. 2019;17(3):3–9.

[12] Andel SA, Pindek S, Spector PE (2016). Being called to safety: occupational callings and safety climate in the emergency medical services. J Occup Environ Med.

[13] Bitan Y, Moran P, Harris J. Evaluating safety culture changes over time with the Emergency Medical Services Safety Attitudes Questionnaire. Aust J Paramed 2019;16.

[14] Patterson PD, Huang DT, Fairbanks RJ, Wang HE (2010). The emergency medical services safety attitudes questionnaire. Am J Med Qual.

[15] Patterson PD, Huang DT, Fairbanks RJ, Simeone S, Weaver M, Wang HE (2010). Variation in emergency medical services workplace safety culture. Prehosp Emerg Care.

[16] Weaver MD, Wang HE, Fairbanks RJ, Patterson D (2012). The association between EMS workplace safety culture and safety outcomes. Prehosp Emerg Care.

[17] Sexton JB, Helmreich RL, Neilands TB, Rowan K, Vella K, Boyden J (2006). The Safety Attitudes Questionnaire: psychometric properties, benchmarking data, and emerging research. BMC Health Serv Res.

[18] Eysenbach G (2004). Improving the quality of Web surveys: the Checklist for Reporting Results of Internet E-Surveys (CHERRIES). J Med Internet Res..

[19] Varricchio CG (2004). Measurement issues concerning linguistic translations. Instrum Clin Health Care Res.

[20] Ministry of Social Affairs and Health, Finland. Health Care Act, 1326:2010. 2013.

[21] Ministry of Social Affairs and Health, Finland. Decree of prehospital emergency care, 585:2017. 2017.

[22] Kosinski M, Matz SC, Gosling SD, Popov V, Stillwell D (2015). Facebook as a research tool for the social sciences: opportunities, challenges, ethical considerations, and practical guidelines. Am Psychol.

[23] Thornton L, Batterham PJ, Fassnacht DB, Kay-Lambkin F, Calear AL, Hunt S (2016). Recruiting for health, medical or psychosocial research using Facebook: systematic review. Internet Interv.

[24] The ethical principles of research with human participants and ethical review in the human sciences in Finland. https://tenk.fi/sites/tenk.fi/files/Ihmistieteiden_eettisen_ennakkoarvioinnin_ohje_2019.pdf: Finnish National Board on Research Integrity; 2019. Accessed: 4 Dec 2020.

[25] Hughes J, Hunter D, Sheehan M, Wilkinson S, Wrigley A. European textbook on ethics in research. Publications Office of the European Union; 2010.

[26] nternational Sociological Association, Faculty of Political Sciences and Sociology, University Complutense; 2001. Code of ethics. https://www.isa-sociology.org/en/about-isa/code-of-ethics. Accessed: 4 Dec 2020.

[27] Byrne BM (2016). Structural equation modeling with AMOS: basic concepts, applications, and programming.

[28] Raykov T, Marcoulides GA. A first course in structural equation modeling (2nd) Lawrence Erlbaum Associates Inc., New Jersey. 2006.

[29] Eysenbach G, Wyatt J (2002). Using the internet for surveys and health research. J Med Internet Res.

[30] Acuna E, Rodriguez C (2004). The treatment of missing values and its effect on classifier accuracy. Classification, clustering, and data mining applications.

[31] Mishra P, Pandey CM, Singh U, Gupta A, Sahu C, Keshri A (2019). Descriptive statistics and normality tests for statistical data. Ann Card Anaesth.

[32] Joseph F, Hair J, Black W, Babin B, Anderson R. Multivariate data analysis: a global perspective. 7th (Global Edition) 2010. Upper Saddle River, NJ: Pearson, Prentice Hall; 2009.

[33] Peterson RA (1994). A meta-analysis of Cronbach's coefficient alpha. J Consum Res.

[34] Bondevik GT, Hofoss D, Husebø BS, Deilkås ECT (2019). The safety attitudes questionnaire–ambulatory version: psychometric properties of the Norwegian version for nursing homes. BMC Health Serv Res.

[35] Göras C, Wallentin FY, Nilsson U, Ehrenberg A (2013). Swedish translation and psychometric testing of the safety attitudes questionnaire (operating room version). BMC Health Serv Res.

[36] Haerkens MH, van Leeuwen W, Sexton JB, Pickkers P, van der Hoeven JG (2016). Validation of the Dutch language version of the Safety Attitudes Questionnaire (SAQ-NL). BMC Health Serv Res.

[37] Gambashidze N, Hammer A, Ernstmann N, Manser T. Psychometric properties of the Georgian version of the Safety Attitudes Questionnaire: a cross-sectional study. BMJ Open. 2020;10(2). 10.1136/bmjopen-2019-034863 PMC7045008 32060162

[38] Noort MC, Reader TW, Shorrock S, Kirwan B (2016). The relationship between national culture and safety culture: implications for international safety culture assessments. J Occup Organ Psychol.

[39] Reader TW, Noort MC, Shorrock S, Kirwan B (2015). Safety sans frontières: An international safety culture model. Risk Anal.

[40] Yorio PL, Edwards J, Hoeneveld D (2019). Safety culture across cultures. Saf Sci.

[41] Zohar D, Luria G (2005). A multilevel model of safety climate: cross-level relationships between organization and group-level climates. J Appl Psychol.

[42] Zohar D (2010). Thirty years of safety climate research: reflections and future directions. Accid Anal Prev.

[43] Edmonson A (2004). Psychological safety, trust, and learning: a group-level lens. Trust and distrust in organizations: dilemmas and approaches.

[44] Berry JC, Davis JT, Bartman T, Hafer CC, Lieb LM, Khan N (2020). Improved safety culture and teamwork climate are associated with decreases in patient harm and hospital mortality across a hospital system. J Patient Saf.

[45] Hall LH, Johnson J, Watt I, O’Connor DB (2019). Association of GP wellbeing and burnout with patient safety in UK primary care: a cross-sectional survey. Br J Gen Pract.

[46] Baier N, Roth K, Felgner S, Henschke C (2018). Burnout and safety outcomes-a cross-sectional nationwide survey of EMS-workers in Germany. BMC Emerg Med.

[47] Koivulahti O, Tommila M, Haavisto E (2020). The accuracy of preliminary diagnoses made by paramedics—a cross-sectional comparative study. Scand J Trauma Resusc Emerg Med.

[48] Aiken LH, Clarke SP, Cheung RB, Sloane DM, Silber JH (2003). Educational levels of hospital nurses and surgical patient mortality. JAMA.

[49] Lefever S, Dal M, Matthiasdottir A (2007). Online data collection in academic research: advantages and limitations. Br J Edu Technol.

[50] Heiervang E, Goodman R (2011). Advantages and limitations of web-based surveys: evidence from a child mental health survey. Soc Psychiatry Psychiatr Epidemiol.

[51] Ministry of Social Affairs and Health, Finland. Medical Research Act, 488/1999; 1999.

