# Supplementary material for: Exploring safety culture in the Finnish ambulance service with Emergency Medical Services Safety Attitudes Questionnaire
Source: Scand J Trauma Resusc Emerg Med. 2021 Oct 12;29:148. doi: 10.1186/s13049-021-00960-9 (PMC8507218; doi:10.1186/s13049-021-00960-9)
Supplement: Supplementary file 1 — Additional file 1. EMS-SAQ and questions/ domain [14, 15] [file 13049_2021_960_MOESM1_ESM.docx]

Additional file 1. EMS-SAQ and questions/ domain (14, 15)

| **Question** | **Domain** |
| --- | --- |
| 1. I like my job | JS1 |
| 1. EMS Personnel input is well-received in this EMS agency | TWC1 |
| 1. I would feel safe being treated by this EMS agency as a patient | SC1 |
| 1. Medical errors are handled appropriately at this EMS agency | SC2 |
| 1. This EMS agency does a good job of training new personnel | WC1 |
| 1. Working at this EMS agency is like being a part of a large family | JS2 |
| 1. The management of this EMS agency supports my daily efforts | POM1 |
| 1. I receive appropriate feedback about my performance | SC3 |
| 1. In this EMS agency, it is difficult to discuss errors | SC4 * |
| 1. Staff turnover at this agency is high |  |
| 1. This EMS agency is a good place to work | JS3 |
| 1. Management does not knowingly compromise the safety of patients | POM2 |
| 1. The levels of staffing at this EMS agency are sufficient to handle the number of calls | POM3 |
| 1. I am encouraged by my colleagues to report any patient safety concerns I may have | SC5 |
| 1. The culture at this EMS agency makes it easy to learn from the errors of others | SC6 |
| 1. This EMS agency deals constructively with problem personnel | WC2 |
| 1. At this EMS agency, it is difficult to speak up if I perceive a problem with patient care | TWC2 * |
| 1. When my workload becomes excessive, my performance is impaired | SR1 |
| 1. I am provided with adequate, timely information about events that might affect my work | POM4 |
| 1. Many EMS personnel at this agency have other full-time or part-time job |  |
| 1. I have seen others make errors that had the potential to harm patients |  |
| 1. I know the proper channels to direct questions regarding patient safety | SC7 |
| 1. I am proud to work at this EMS agency | JS4 |
| 1. Disagreements at this EMS agency are resolved appropriately (i.e., not who is right, but what is best for the patient) | TWC3 |
| 1. I am less effective at work when fatigued | SR2 |
| 1. I am more likely to make errors in tense or hostile situations | SR3 |
| 1. I have the support I need from other personnel to care for patients | TWC4 |
| 1. It is easy for personnel at this EMS agency to ask questions when there is something that they don’t understand | TWC5 |
| 1. Personnel here work together as a well-coordinated team | TWC6 |
| 1. I have the co-workers who are actively looking for additional full-time or part-time work |  |
| 1. Morale at this EMS agency is high | JS5 |
| 1. Trainees in my discipline are adequately supervised | WC3 |
| 1. I have made errors that had the potential to harm patients |  |
| 1. Fatigue impairs my performance during emergency situations | SR4 |
| 1. During emergency situations (e.g. cardiac arrests, traumas etc.) my performance is not affected by working with inexperienced or less capable personnel |  |
| 1. Personnel frequently disregard rules or guidelines (e.g. treatment protocols, standard operating procedures, etc.) that are established for this EMS agency |  |
| 1. A confidential reporting system is helpful for improving patient safety |  |
| 1. I may hesitate to use a reporting system because I am concerned about being identified |  |
| 1. This agency provides me with the training to prevent ambulance driving accidents |  |
| 1. I have co-workers who are actively looking to leave this agency for other employment |  |
| 1. This agency could do more to improve emergency vehicle driver safety |  |
| 1. When moving a patient, I have the training to prevent injury to the patient |  |
| 1. When moving a patient, I have the right equipment to prevent injury to the patient |  |
| 1. All the necessary information for treating patients is routinely available to me | WC4 |
| 1. Patient safety is constantly reinforced as the priority in this EMS agency |  |
| 1. Emergency vehicle or aircraft accidents occurs at this EMS agency |  |
| 1. Emergency vehicle or aircraft accident close-calls (near-misses) occur at this EMS agency |  |
| 1. Patient handling mishaps (e.g. stretcher collapse, patient drop or fall, etc.) occur at this EMS agency |  |
| 1. Medical adverse events (incidents where a patient was harmed from medical care or medical equipment malfunction) occur at this EMS agency |  |
| 1. Medical adverse event close-calls (near-misses) occur at this EMS agency |  |
| SC = Safety climate  TWC = Teamwork climate  POM = Perceptions of management  SR = Stress Recognition  WC = Working conditions  JS = Job satisfaction  * = Reverse coded in the analysis to match the other questions |  |
