# Supplementary material for: Exploring safety culture in the Finnish ambulance service with Emergency Medical Services Safety Attitudes Questionnaire
Source: Scand J Trauma Resusc Emerg Med. 2021 Oct 12;29:148. doi: 10.1186/s13049-021-00960-9 (PMC8507218; doi:10.1186/s13049-021-00960-9)
Supplement: Supplementary file 2 — Additional file 2. Percentage of positive responses between the respondent’s characteristic [file 13049_2021_960_MOESM2_ESM.docx]

Additional file 2. Percentage of positive responses between the respondent’s characteristic

| **Characteristic (n)** | **Safety climate** | **Teamwork climate** | **Perceptions of management** | **Stress recognition** | **Working conditions** | **Job satisfaction** |
| --- | --- | --- | --- | --- | --- | --- |
| Percentage of positive responses (mean > 75) | | | | | | |
| **Total n=327** | 25.4 | 26.9 | 27.2 | 37.3 | 18.7 | 51.1 |
| **Gender n=324 *** | NS | NS | NS | NS | NS | NS |
| Female (161) | 23.6 | 26.1 | 31.1 | 39.1 | 19.3 | 49.7 |
| Male (163) | 27.0 | 27.6 | 23.3 | 35.6 | 17.8 | 52.1 |
| **Age n=273 **** | NS | NS | NS | NS | NS | NS |
| ≤ 25 (80) | 34.8 | 21.7 | 13.0 | 39.1 | 17.4 | 56.5 |
| 26-30 (79) | 21.8 | 20.5 | 25.6 | 38.5 | 15.4 | 50.0 |
| 31-35 (71) | 22.5 | 25.4 | 23.9 | 35.2 | 18.3 | 42.3 |
| 36-40 (53) | 17.0 | 26.4 | 20.8 | 47.2 | 17.0 | 52.8 |
| 41-45 (31) | 33.3 | 30.0 | 33.3 | 33.3 | 20.0 | 53.3 |
| ≥ 46 (19) | 33.3 | 27.8 | 38.9 | 38.9 | 27.8 | 44.4 |
| **Education level n=327 **** | NS | NS | NS | NS | NS | NS |
| Master’s (50) | 22.0 | 20.0 | 20.0 | 42.0 | 16.0 | 40.0 |
| Bachelor’s (225) | 24.4 | 27.6 | 28.4 | 39.1 | 17.3 | 51.1 |
| Vocational (46) | 34.8 | 34.8 | 30.4 | 23.9 | 30.4 | 65.2 |
| Other (6) | 16.7 | 0.0 | 16.7 | 33.3 | 0.0 | 33.3 |
| **Working experience n=327 **** | NS | NS | NS | NS | NS | NS |
| ≤ 5 years (110) | 27.3 | 26.4 | 30.0 | 39.1 | 15.5 | 58.2 |
| 6-10 years (113) | 25.7 | 29.2 | 25.7 | 33.6 | 20.4 | 46.9 |
| 11-15 years (53) | 20.8 | 18.9 | 17.0 | 41.5 | 13.2 | 43.4 |
| > 15 years (51) | 25.5 | 31.4 | 35.3 | 37.3 | 27.5 | 52.9 |
| **Position type**  **n=325 *** | NS | NS | NS | NS | p = 0.043 | NS |
| Advanced level (264) | 24.6 | 26.5 | 26.9 | 39.4 | 16.7 | 49.6 |
| Basic level (61) | 29.5 | 29.5 | 29.5 | 27.9 | 27.9 | 57.4 |
| **Employment status n=327 *** | p = 0.037 | NS | NS | NS | NS | NS |
| Full-time (295) | 23.7 | 26.4 | 26.1 | 38.3 | 17.6 | 49.5 |
| Part-time (32) | 40.6 | 31.3 | 37.5 | 28.1 | 28.1 | 65.6 |
| **Shift type n=327 **** | NS | p = 0.009 | p = 0.000 | NS | NS | NS |
| 24-hour shifts (131) | 28.2 | 35.9 | 39.7 | 36.6 | 21.4 | 58.8 |
| Two shift (181) | 24.3 | 21.5 | 17.7 | 35.4 | 18.2 | 46.4 |
| Mix (24h+12h and/or 8h) (15) | 13.3 | 13.3 | 33.3 | 66.7 | 0.0 | 40.0 |
| **Affiliation n=327 **** | NS | NS | p = 0.009 | NS | p = 0.032 | NS |
| Health care district (161) | 24.8 | 21.7 | 19.9 | 38.5 | 13.7 | 46.6 |
| Rescue department (119) | 24.4 | 30.3 | 32.8 | 38.7 | 26.1 | 58.0 |
| Private (47) | 29.8 | 36.2 | 38.3 | 29.8 | 17.0 | 48.9 |
| **Catchment area for highly responsive care n=327 **** | NS | p = 0.002 | p = 0.000 | NS | p = 0.016 | p = 0.004 |
| Helsinki University Hospital (112) | 29.5 | 20.5 | 23.2 | 41.1 | 18.8 | 50.0 |
| Turku University Hospital (41) | 29.3 | 39.0 | 34.1 | 39.0 | 19.5 | 56.1 |
| Tampere University Hospital (50) | 16.0 | 26.0 | 30.0 | 38.0 | 10.0 | 48.0 |
| Kuopio University Hospital (63) | 15.9 | 15.9 | 6.3 | 27.0 | 11.1 | 34.9 |
| Oulu University Hospital (61) | 32.8 | 42.6 | 49.2 | 39.3 | 32.8 | 68.9 |

* p-values (not corrected) counted with *X^2^-* test

** p-values (not corrected) counted with Fisher’s exact test
